# Molecular diagnosis of bird-mediated pest consumption in tropical farmland

**DOI:** 10.1186/2193-1801-3-630

**Published:** 2014-10-24

**Authors:** Daniel S Karp, Seth Judson, Gretchen C Daily, Elizabeth A Hadly

**Affiliations:** Department of Biology, Stanford University, Stanford, CA 94305 USA; Center for Conservation Biology, Stanford University, Stanford, CA 94305 USA; Woods Institute for the Environment, Stanford University, Stanford, CA 94305 USA; Now at Department of Environmental Science, Policy, and Management, University of California Berkeley, Berkeley, CA 94720 USA

**Keywords:** Agriculture, Avian, Coffee berry borer beetle, DNA barcoding, Molecular scatology

## Abstract

Biodiversity loss will likely have surprising and dramatic consequences for human wellbeing. Identifying species that benefit society represents a critical first step towards predicting the consequences of biodiversity loss. Though natural predators prevent billions of dollars in agricultural pest damage annually, characterizing which predators consume pests has proven challenging. Emerging molecular techniques may illuminate these interactions. In the countryside of Costa Rica, we identified avian predators of coffee’s most damaging insect pest, the coffee berry borer beetle (Coleoptera:Scolytidae *Hypothenemus hampeii*), by assaying 1430 fecal samples of 108 bird species for borer DNA. While feeding trials confirmed the efficacy of our approach, detection rates were low. Nevertheless, we identified six species that consume the borer. These species had narrow diet breadths, thin bills, and short wings; traits shared with borer predators in other systems. Borer predators were not threatened; therefore, safeguarding pest control necessitates managing species beyond those at risk of regional extinction by maintaining populations in farmland habitats. Generally, our results demonstrate potential for pairing molecular methods with ecological analyses to yield novel insights into species interactions.

**Electronic supplementary material:**

The online version of this article (doi:10.1186/2193-1801-3-630) contains supplementary material, which is available to authorized users.

## Introduction

Managing the benefits people receive from nature, or ecosystem services, requires a detailed understanding of ecosystem processes. In particular, biodiversity-driven services, such as pest control on farms, requires knowledge of cropping systems, the habitats in and around croplands, and the interactions among the many organisms that inhabit them. Interactions are complex and often change over space and time (Luck et al. 2003); therefore, a critical first step is identifying the species and populations that provide benefits to society (Kremen 2005). Identifying service providers, however, may not be straightforward. For example, predation is rarely witnessed directly, making it difficult to identify the predators of crop pests. Pest control is a critical service; in the United States, insect predators save farmers billions of dollars annually in avoided pest damage (Losey and Vaughan 2006). Several different techniques have been utilized to identify predator–prey interactions. An indirect approach is using stable isotopes to determine trophic positions (Kelly 2000). A direct approach for identifying predators is visual identification of prey remains in predators’ guts or feces (Leelapaibul et al. 2005). While visual identification of prey gut contents can sometimes yield the necessary taxonomic resolution to identify insect pests, the necessary inspection labor is considerable and sampling techniques often result in high mortality rates among study subjects.

Molecular identification techniques, however, offer great potential to yield insight into predator–prey interactions (Symondson 2002; King et al. 2008; Pompanon et al. 2012). These techniques often rely on targeting and sequencing a standardized DNA region across species to facilitate identifications (Valentini et al. 2009). Applications of this approach are diverse; for example, detecting diet shifts in ancient humans (Adler et al. 2013), characterizing biological communities in hydrothermal vents (Sogin et al. 2006), identifying illegal trade in endangered species (Domingo-Roura et al. 2006), and surveying rare mammals with DNA from leeches (Schnell et al. 2012). Similarly, molecular identification in feces, regurgitate, and stomach contents from carnivores, insectivores, and herbivores of diverse taxa has been used to infer diet (Deagle et al. 2005; Clare et al. 2009; Pegard et al. 2009; King et al. 2011; Jedlicka et al. 2013). While the application of molecular diet analysis is becoming widespread, the technique is not without limitations. First, predators vary in gut retention times and digestion processes, which may affect detection rates and complicate comparisons among species (Afik and Karasov 1995; Markman et al. 2006). Second, DNA assays can misattribute diet in the presence of intraguild predation— that is, if the DNA of the prey of an intermediate predator is found in the fecal samples of a top predator (secondary predation) (King et al. 2008). Finally, digestion degrades prey DNA, making fecal analysis more sensitive than other PCR procedures to DNA quantity (King et al. 2008).

Despite these shortcomings, several studies have used molecular techniques to identify suites of pest predators, largely through DNA analysis of arthropod predators’ gut contents (Fournier et al. 2008; King et al. 2011; Boreau de Roincé et al. 2012). Less work has focused on vertebrate insectivores, despite their great potential to control pest infestations (Cleveland et al. 2006; Karp et al. 2013; Maas et al. 2013). Those that have studied vertebrate predators of insect pests tend to analyze single predator species rather than communities (Clare et al. 2011; McCracken et al. 2012). Further, analyses have neglected the biologically diverse, tropical countries that may stand to benefit most from conservation-minded pest-management plans (Jaramillo et al. 2010).

We used molecular fecal analysis to identify bird predators of coffee’s most damaging insect pest— the coffee berry borer beetle (Coleoptera:Scolytidae *Hypothenemus hampeii*). Coffee is cultivated across the tropics, with a total export value over US$20 billion and twenty million households involved in its production (International Coffee Organization 2012). The borer has invaded almost every coffee-producing country in recent years. In fact, the borer invaded Costa Rica in 2000 (Staver et al. 2001) and our study sites in 2005. It spends the majority of its lifecycle within coffee berries, overwintering in un-harvested berries and undergoing a major dispersal event several months after the first rains (Damon 2000). Previous exclusion experiments have shown that birds consume the borer, likely during the primary dispersal event or secondary movements to adjacent berries throughout the year (Kellermann et al. 2008; Karp et al. 2013). The borer’s small size (~2 mm) makes directly witnessing predation unlikely (Damon 2000; Karp et al. 2013).

Our work builds on Karp et al. (2013), which used exclosures to quantify bird-mediated borer control. Here, we sought to characterize more completely which species are borer predators, supplementing their analysis with an additional 961 fecal samples and 33 bird species (for a total of 1430 samples across 108 species). In addition, we verified this approach through feeding trials with three insectivorous bird species. Finally, we compiled a database of bird conservation and functional traits to make a preliminary determination of the traits associated with borer consumption and to assess whether species that important for controlling damaging insect pests are also conservation targets.

## Materials and methods

### Study site and sample collection

Our investigation centered on coffee plantations in southern Costa Rica, near the Las Cruces Biological Station of the Organization for Tropical Studies. We worked on two coffee plantations—a small (30 ha) family plantation and a large (250 ha) commercial operation. Both are situated at ~1100 m asl, and cultivate coffee (*Coffea arabica*) under sun.

We collected fecal samples from birds in April-May of 2010 and 2011, when borers were at peak dispersal. All animals were treated humanely, in accordance with the Institutional Animal Care and Use Committee (IACUC) guidelines and approved by the Administrative Panel on Laboratory Animal Care (APLAC) of Stanford University (Assurance Number A3213-01; Protocol ID 26920). We placed three mist-netting stations at each plantation and visited each station three times per year. Each station was composed of 20 12 m × 2.5 m mist nets, located between rows of coffee and within patches of forest next to plantations. Our surveys began at sunrise, continuing for 5–6 hours until bird activity subsided. All birds were placed in breathable cotton bags until they could be identified and uniquely marked with a metal leg band. After release, bags were checked for feces, which were removed with ethanol-flamed tweezers and placed in ready glass vials or 2 ml Eppendorf tubes filled with ethanol. They were refrigerated until transport to the United States and then stored at −20°C. Bags were always immersed in a 10% bleach solution, sun-dried, and washed after use.

We conducted feeding trials with three common, small insectivores that frequent coffee plantations and were expected to consume the borer: Rufous-capped Warbler (Passeriformes: Parulidae *Basileuterus rufifrons*), Rufous-breasted Wren (Passeriformes: Troglodytidae *Pheugopedius rutilus*), and Plain Wren (Passeriformes: Troglodytidae *Cantorchilus modestus*). Individuals were fed 0, 2, 4, or 8 borers collected in nearby plantations (0 borers: n = 24 fecal samples from 9 individuals; 2 borers: n = 26 samples, 13 individuals; 4 borers: n = 34 samples, 21 individuals; 8 borers: n = 26 samples, 9 individuals). Specifically, we held each bird’s mouth open and placed the borers inside with a sterilized tweezers. We then used a syringe to inject water into the bird’s mouth, inducing it to swallow the insects. Birds were then placed in mesh cages over sterilized cotton floors. Cages were checked for fecal samples every 15 minutes for 1.5 hours; stressed birds were released earlier. Though referenced in Karp *et al.* (2013), feeding trial data were not previously analyzed.

### Molecular methods

We poured off ethanol and weighed samples prior to DNA extraction. For all species that were strictly frugivorous, we combined samples derived from different individuals of the same species captured at the same plantation to reduce processing time and cost. The combined sample was homogenized and a <0.25 g subset was extracted. Because many more individuals were captured, in the second year we additionally combined samples from multiple individuals of the same species at the same plantation for all omnivores and large insectivores. Again, samples were homogenized and a <0.25 g subset was extracted. Samples from feeding trials and from small insectivores were always extracted individually. Extraction was performed with kits (QIAamp DNA Stool Kit, Qiagen, California, United States), modified to increase product yield (Zeale et al. 2010). All extractions were accompanied with negative controls with no fecal material added so that we could identify any possible sources of contamination.

Following extraction, we amplified borer DNA through PCR with borer-specific primers (Jaramillo et al. 2010). We targeted an 185 bp segment of mitochondrial cytochrome oxidase I (COI). We used 12.2 μL reactions composed of 8.2 μL deionized water, 0.3 μL 10 μg/μL BSA, 0.45 μL 10 mM dNTPs, 1.41 μL 10x PCR buffer, 0.61 μL 50 mM MgCl_2_, 0.28 μL 20 mM forward primer, 0.28 μL 20 mM reverse primer, 0.06 μL 5U/L Taq Platinum (Life Technologies, Invitrogen, California, USA), and 0.6 μL template DNA. Because DNA degrades in the gut, our protocol consisted of a high number of cycles; specifically, a 2-min denaturation at 95°C, followed by 50 cycles of 22 s at 94°C, 22 s at 55°C and 30 s at 72°C, and a final incubation of 8 min at 72°C. Each PCR reaction was accompanied with negative and positive controls, derived from feeding trial samples. Though primers were designed to be borer specific, we sent all amplicons of the expected size range for sequencing (Elim Biopharm, Hayward, California, USA) because many PCR cycles can result in amplification of non-target DNA. We used Sequencher (Genes Code Corporation, Ann Arbor, MI) to form consensus alignments of DNA reads from forward and reverse primers that were then compared to a borer reference sequence. Only sequences with clean, discernable peaks at target base pairs were analyzed. Those sequences with >98% similarity to borer reference sequences were deemed successful borer identifications. The next most similar sequence from another species in Genbank at the target amplicon was 85% similar.

We accidentally contaminated several samples (n = 80) with PCR amplicons, necessitating the development of alternate primers. We amplified an 113 bp segment of COI, outside the previous amplification region, with forward (5′- GATCAGTAAAAATTACAGCAATCT -3′) and reverse (5′-TCATTTTTTGACCCTGCC-3′) primers. Reactions were carried out using the same reagents and protocols, apart from the annealing step (45°C rather than 55°C). Products were visualized on gels, and negative controls confirmed that the contamination was previous PCR product. Because primers were not borer-specific, all products of the expected size range were sequenced and compared to reference sequences. After the borer, the next most similar sequence in Genbank was 86% similar.

### Bird functional traits

We assessed whether confirmed borer predators shared functional traits through compiling a trait database for birds in our study area, focusing on resource and acquisition traits that may affect pest-control provision (Flynn et al. 2009; Luck et al. 2012). We used measurements from birds we captured, and a bird population dynamics dataset collected at 18 nearby sites (Mendenhall et al. 2011). Wing chord length and mass were obtained from the population dynamics dataset. We also calculated the total number of captures for each species. We collected bill width (at nares), bill length (culmen), and tarsus length from species that we trapped during fecal sample collection. Body lengths were obtained from literature (Stiles and Skutch 1989).

We gathered behavioral traits from literature (Stiles and Skutch 1989). We translated foraging stratum into an ordinal scale (1-ground; 2-lower vegetation; 3-middle vegetation; 4-sub canopy; 5-canopy; 6-above canopy), and calculated the average foraging stratum for each species. We quantified diet breadth as the number of food categories consumed (fruit, nectar, seeds, crops, grit/leaves, invertebrates, vertebrates, and aquatic organisms). From literature (Stiles and Skutch 1989) and conversations with local ornithologists (C.D. Mendenhall, F.O. Brenes, J. Zook), we also identified species that consumed insects and the subset that specialized strictly on insects.

### Analysis

We used Generalized Linear Mixed Models (GLMM) to identify variables that significantly influenced the probability of borer DNA detection in feeding trials (Zuur et al. 2009). The model contained a logit link and binomial error structure, and the feeding trial as a random effect. Species identity, elapsed time since feeding, number of borers fed, fecal sample mass, and 2-way interactions (excluding those with species identity) were included as predictors. We then used backwards model selection, iteratively dropping predictor variables and comparing nested models with Aikaike Information Criteria and likelihood-ratio tests (Zuur et al. 2009).

Next, we determined whether species confirmed as borer predators shared traits. Because very few of the birds that were not involved feeding trials tested positive for borer DNA, it was impossible to use logistic regression to associate bird traits with borer predation. Instead, we created a randomization procedure in which six species were drawn at random 1000 times from a species pool (the species for which feces were obtained), and the average trait value for these species was calculated each time. This procedure generated a null distribution for each trait that could then be compared to the average trait value of confirmed borer predators. If the observed trait value fell outside the 95% confidence interval, then we determined the trait was a significant predictor of borer-predator identity.

## Results

We amplified and sequenced borer DNA from the feces of six species: Buff-throated Foliage-Gleaner (Passeriformes: Furnariidae *Automolus ochrolaemus*; detected in 1 of 8 samples), Common Tody-Flycatcher (Passeriformes: Tyrannidae *Todirostrum cinereum*; 1 of 4), Rufous-breasted Wren (Passeriformes: Troglodytidae *Pheugopedius rutilus*; 1 of 18), Rufous-capped Warbler (Passeriformes: Parulidae *Basileuterus rufifrons*; 5 of 66), White-tailed Emerald (Apodiformes: Trochilidae *Elvira chionura*; 1 of 2), and Yellow Warbler (Passeriformes: Parulidae *Setophaga petechia*; 1 of 26). The majority of detections (8 of 10) were derived from surveys conducted in 2010 and reported in Karp *et al.* (2013), even though fewer samples were collected in 2010 (478 vs. 961 samples). In total, 30 (Jaramillo *et al.* primers) and 27 (our developed primers) samples yielded PCR products of the expected size range, 4 and 6 of which were >98% sequence similar to borers.

**Figure 1:**
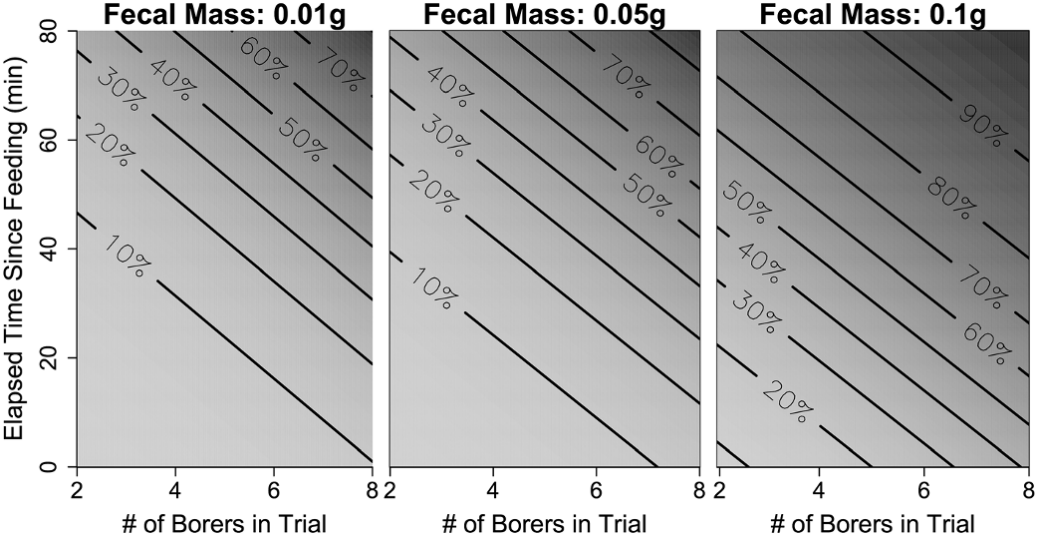
**Predicted percent likelihood of borer detection in fecal samples from feeding trials.** Detection probability increased with the size of fecal sample, elapsed time since feeding, and number of borer beetles in the feeding trial. Panels depict predicted percent likelihood of detection from GLMMs, and predictions for fecal samples of 0.01 g, 0.05 g, and 0.1 g.

Though detections rates were low, feeding trials confirmed the efficacy of our approach. Fifteen samples yielded PCR products of the expected size range, all of which were confirmed as borers through sequencing (13.6% of tested samples). Borer DNA was detected most often when birds were fed more borers and defecated larger fecal samples (Figure 1; Additional file 1: Table S1). Additionally, detection probability increased with elapsed time since feeding. For example, models predicted that, for birds that were fed 4 borers and defecated a 0.05 g fecal pellet, detection probability increased from 10% at 20 minutes after feeding to 50% at 80 minutes after feeding. Species identity of feeding trial birds did not influence detection probability. While it is possible that positive borer detections in feeding trials could have resulted from prior consumption, no detections occurred when birds were not fed borers. Moreover, the low detection rates in non-feeding trial birds further reduce the likelihood that positive detections were the result of prior foraging.

**Figure 2:**
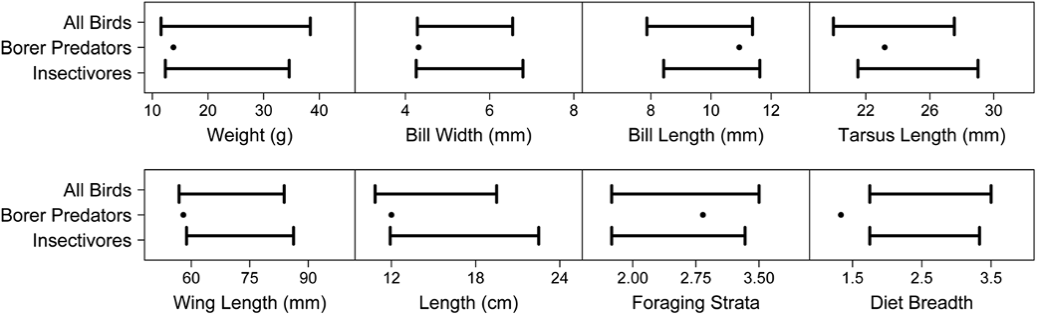
**Functional trait analyses of confirmed borer predators.** Borer predators had narrower bills, marginally shorter wings, and were more specialized in diet than other surveyed species. They were also marginally smaller in weight and length. Plots depict 95% confidence intervals of mean trait values from 1000 randomizations in which species were drawn from a pool of all surveyed species (top line and whiskers) or insectivores only (bottom line and whiskers). Points depict average trait values for the six confirmed borer predators. For actual functional trait values of the six predators, see Additional file 1: Figure S1.

We found that functional traits differed between confirmed borer predators and other sampled species (Figure 2; Additional file 1: Figure S1). Borer predators had narrower bills (Two-tail *P* = 0.06; One-tail *P* = 0.03) and shorter wing chords (Two-tail *P* = 0.09; One-tail *P* = 0.05) than expected. Though not significant, predators also tended to be smaller, both in mass (Two-tail *P* = 0.18; One-tail *P* = 0.09) and length (Two-tail *P* = 0.13; One-tail *P* = 0.26). Diets were specialized (Two-tail *P* < 0.01; One-tail *P* < 0.01), and insectivores were overly represented (*P* = 0.05)— only the White-tailed Emerald was not a specialized insectivore. Borer predators were not species of general conservation concern. Predators were equally abundant (*P* = 0.51) to other species in our study system, and were neither endemic nor listed on the IUCN red list. Leveraging traits that were over-represented in confirmed borer predators, we predicted other species that may consume the borer but no pest DNA was found in their fecal samples, likely the result of low detection rates (Table 1).

**Table 1:** **Traits of possible borer-consuming birds**

English name	Scientific name	Mass (g)	Beak wid. (mm)	Beak len. (mm)	Tarsus (mm)	Wing (mm)	Len. (cm)	Strata	Diet breadth	Total capt.
***American Redstart***	***Setophaga ruticilla***	***7***	4.5*	9.1*	17.9*	***60***	***12***	***3***	***1***	***14***
***Black-and-white Warbler***	***Mniotilta varia***	***10***	***3***	***9***	17*	***64***	***13***	***4***	***1***	***25***
***Black-throated Blue Warbler***	***Dendroica caerulescens***	10	NA	9.4*	18.1*	65.2*	12	4	1	0
Buff-rumped Warbler	Phaeothlypis fulvicauda	15	5	9	NA	64	13	1	1	93
Canada Warbler	Wilsonia canadensis	9	3	6	22	61	12	2	1	0
Chestnut-sided Warbler	Dendroica pensylvanica	9	4	7	18.3*	62	12	4	2	118
Common Yellowthroat	Geothlypis trichas	10	NA	10*	21*	56*	68	2	1	0
Golden-winged Warbler	Vermivora chrysoptera	9	NA	8.2*	17*	60	12	4	1	14
Kentucky Warbler	Oporornis formosus	14	NA	8.5*	21.5*	67*	13	1	1	34
MacGillivray’s Warbler	Oporornis tolmiei	12	3.0*	7.6*	20.7*	60	12	1	1	3
Masked Yellowthroat	Geothlypis aequinoctialis	14	NA	NA	NA	56	14	2	1	5
Mourning Warbler	Oporornis philadelphia	11	4	8	24	59	12	1	1	168
***Northern Parula***	***Parula americana***	8	NA	7.4*	14.9*	57.9*	11	5	1	0
Pale-breasted Spinetail	Synallaxis albescens	14	3	8	22	50	14	2	1	8
Plain Antvireo	Dysithamnus mentalis	14	5	9	NA	59	12	2	1	30
Plain Xenops	Xenops minutus	12	4	10	NA	62	12	3	1	22
***Prairie Warbler***	***Dendroica discolor***	7.6	3.2*	10.1*	18.2*	56*	11	NA	1	0
Red-faced Spinetail	Cranioleuca erythrops	16	NA	NA	NA	65	15	3	2	6
**Rufous-breasted Wren**	**Thryothorus rutilus**	**16**	**4**	**11**	**24**	**56**	**13**	**3**	**1**	**74**
**Rufous-capped Warbler**	**Basileuterus rufifrons**	**11**	**5**	**8**	**23**	**54**	**13**	**3**	**2**	**152**
Scale-crested Pygmy-Tyrant	Lophotriccus pileatus	8	NA	NA	NA	49	9	4	1	49
Slate-throated Redstart	Myioborus miniatus	9	NA	NA	NA	62	12	5	1	77
Slaty Spinetail	Synallaxis brachyura	18	4	10	25	54	15	2	1	12
Spotted Barbtail	Premnoplex brunnescens	16	NA	NA	NA	63	14	3	1	16
White-breasted Wood-Wren	Henicorhina leucosticta	17	4	12	28	56	10	2	1	102
Wilson’s Warbler	Wilsonia pusilla	8	3.0*	6.1*	15.4*	56	11	4	1	17
**Yellow Warbler**	**Dendroica petechia**	**9**	**4**	**8**	**21**	**59**	**12**	**3**	**1**	**134**

Bolded species are borer consumers (italicized confirmed in Jamaica). Others are insectivores encountered in coffee that share traits with borer consumers (mass, beak width, wing, and diet breadth).

Beak Wid. = Beak Width; Beak Len. = Beak Length; Len. = Length; Total Capt. = Total Captures; Evol. Uni. = Evolutionary Uniqueness. *Indicates data from Cornell’s Birds of North America. NA indicates data deficiency.

## Discussion

Ecosystem-service management necessitates identifying service providers, especially in the many agricultural systems that are rapidly expanding and intensifying (Foley et al. 2005). Our analysis of ~1500 fecal samples documented that six Costa Rican bird species consume coffee’s most damaging insect pest. Still, detection rates were very low: only 0.7% of analyzed samples contained borer DNA. We offer several explanations for low detection. First, we sampled the entire bird community, including frugivores which do not likely consume the borer. Second, borer abundance is low in our study system. Only 2.5% of berries across plantations are currently infested with borers, whereas infestation has soared above 90% in other countries (Mugo and Kimemia 2011).

Third, detection windows may be narrow. We detected borer DNA in only one sample defecated within 30 min of feeding. Insect DNA could be detected in Carrion Crow feces 30 minutes to 4 hours after consumption (Oehm et al. 2011). Borers disperse most often and hence are most vulnerable to predation in the afternoon (Damon 2000). Because tropical weather constraints precluded afternoon sampling, a mismatch in sampling and consumption could have depressed detections. Finally, feeding trials demonstrated that false negatives are regular. Models predicted that a positive detection was ~20 times more likely when birds were fed 8 borers and defecated 0.1 g versus 2 borers and 0.01 g. In addition to DNA degradation in the gut, our extraction and PCR procedures may be prone to false negatives. First, PCR inhibitors can persist through extraction and impede DNA amplification from fecal pellets (Jedlicka et al. 2013). Second, unlike the primers developed by Jaramillo *et al*. (2010), the primers that we developed (used on 80 of 1430 samples) were not specific to the berry borer, meaning the primers could have amplified DNA from any one of the many species of insects that a bird had recently consumed. Moreover, iterant non-specific PCR binding of either primer set could generate chimeric sequences of multiple species. Accordingly, only 10 of the 57 samples that yielded PCR products of the expected size range were identified as borer DNA after sequencing. Future work could utilize a post-PCR sorting method such as next generation sequencing or cloning to help reduce the frequency of false negatives (Jedlicka et al. 2013).

Low detection rates suggest that there are other species that consume the borer that we did not identify. The species we did identify, however, shared traits that may be characteristic of these other predators. All identified borer predators except the nectarivorous White-tailed Emerald were strict insectivores. Unsurprising given the borer’s size (~2 mm), borer predators had narrow bills. Additionally, these species had short wings, ideal for navigating the dense coffee understory (Podulka et al. 2004). It is possible that functional traits would change with a larger sample of predators; however, confirmed borer predators in Jamaican coffee plantations shared many of these traits (Table 1), supporting our hypothesis that they may help predict other predators (T. Sherry *Unpublished Data).* A key difference between our studies, however, is that only one of the species that we identified as a borer predator is migratory (Yellow Warbler- *Setophaga petechia*). We collected our fecal samples during the period of maximum borer dispersal (April-June), a time when most migratory species are absent from Costa Rica. Because migratory species could consume borers during their secondary dispersals that occur throughout the year, future work should temporally expand sampling effort to ensure that migratory species are well represented.

Our work yielded the critical management insight that managing the predators of crop pests may require looking beyond traditional conservation targets. The six documented borer predators were not rare, endemic, or listed on the IUCN red list. Traditional conservation efforts for threatened species often center on delineating large protected areas. Focusing conservation explicitly in agricultural landscapes could benefit species involved in providing critical ecosystem services to farmers (*e.g.* through restoring native trees within and surrounding plantations Karp *et al.*^2013^). By confirming that birds consume pests, our work could thus help change attitudes towards biodiversity in human-dominated landscapes by fostering greater recognition of its role in supporting human wellbeing.

Species interactions play a pivotal role in many ecologically and economically important ecosystem processes. Uncovering the basic relationships between animals and their food is critical for managing pest control, pollination, seed dispersal, and sanitation (scavenging). Molecular methods can provide us with a window into these interactions, in some instances for the very first time. Our results demonstrate how identifying just a few key interactions between predators and their prey can yield potential insights for management. Indeed, managing nature to enhance both biodiversity and human wellbeing requires diverse approaches. Techniques and practices have already been borrowed from fields as diverse as agronomy, economics, hydrology, psychology, and sociology. Our results indicate that molecular biology offers ecologists the ability to expand their toolkit in key dimensions and, in turn, advance ecosystem-service management.

## Electronic supplementary material

Additional file 1: **Supplementary Tables and Figures.** (DOCX 299 KB)

## References

[CR1] Adler CJ, Dobney K, Weyrich LS, Kaidonis J, Walker AW, Haak W, Bradshaw CJA, Townsend G, Soltysiak A, Alt KW, Parkhill J, Cooper A (2013). Sequencing ancient calcified dental plaque shows changes in oral microbiota with dietary shifts of the Neolithic and Industrial revolutions. Nat Genet.

[CR2] Afik D, Karasov WH (1995). The trade-offs between digestion rate and efficiency in warblers and their ecological implications. Ecology.

[CR3] Boreau de Roincé C, Lavigne C, Ricard J-M, Franck P, Bouvier J-C, Garcin A, Symondson WOC (2012). Predation by generalist predators on the codling moth versus a closely-related emerging pest the oriental fruit moth: a molecular analysis. Agric For Entomol.

[CR4] Clare EL, Fraser EE, Braid HE, Fenton MB, Hebert PDN (2009). Species on the menu of a generalist predator, the eastern red bat (Lasiurus borealis): using a molecular approach to detect arthropod prey. Mol Ecol.

[CR5] Clare EL, Barber BR, Sweeney BW, Hebert PDN, Fenton MB (2011). Eating local: influences of habitat on the diet of little brown bats (Myotis lucifugus). Mol Ecol.

[CR6] Cleveland CJ, Betke M, Federico P, Frank JD, Hallam TG, Horn J, Lopez JD, McCracken GF, Medellín RA, Moreno-Valdez A, Sansone CG, Westbrook JK, Kunz TH (2006). Economic value of the pest control service provided by Brazilian free-tailed bats in south-central Texas. Front Ecol Environ.

[CR7] Damon A (2000). A review of the biology and control of the coffee berry borer, Hypothenemus hampei (Coleoptera: Scolytidae). Bull Entomol Res.

[CR8] Deagle BE, Tollit DJ, Jarman SN, Hindell MA, Trites AW, Gales NJ (2005). Molecular scatology as a tool to study diet: analysis of prey DNA in scats from captive Steller sea lions. Mol Ecol.

[CR9] Domingo-Roura X, Marmi J, Ferrando A, López-Giráldez F, Macdonald DW, Jansman HAH (2006). Badger hair in shaving brushes comes from protected Eurasian badgers. Biol Conserv.

[CR10] Flynn DFB, Gogal-Prokurat M, Molinari N, Richers BT, Lin BB, Simpson N, Mayfield M, DeClerck F (2009). Loss of functional diversity under land use intensification across multiple taxa. Ecol Lett.

[CR11] Foley JA, Defries R, Asner GP, Barford C, Bonan G, Carpenter SR, Chapin FS, Coe MT, Daily GC, Gibbs HK, Helkowski JH, Holloway T, Howard EA, Kucharik CJ, Monfreda C, Patz JA, Prentice IC, Ramankutty N, Snyder PK (2005). Global consequences of land use. Science.

[CR12] Fournier V, Hagler J, Daane K, de León J, Groves R (2008). Identifying the predator complex of Homalodisca vitripennis (Hemiptera: Cicadellidae): a comparative study of the efficacy of an ELISA and PCR gut content assay. Oecologia.

[CR13] International Coffee Organization (2012). Annual Review.

[CR14] Jaramillo J, Chapman EG, Vega FE, Harwood JD (2010). Molecular diagnosis of a previously unreported predator–prey association in coffee: Karnyothrips flavipes Jones (Thysanoptera: Phlaeothripidae) predation on the coffee berry borer. Naturwissenschaften.

[CR15] Jedlicka JA, Sharma AM, Almeida RPP (2013). Molecular tools reveal diets of insectivorous birds from predator fecal matter. Conserv Genet Resour.

[CR16] Karp DS, Mendenhall CD, Sandi RF, Chaumont N, Ehrlich PR, Daily GC (2013). Forest bolsters bird abundance, pest control, and coffee yield. Ecol Lett.

[CR17] Kellermann JL, Johnson MD, Stercho AM, Hackett SC (2008). Ecological and economic services provided by birds on Jamaican Blue Mountain coffee farms. Conserv Biol.

[CR18] Kelly JF (2000). Stable isotopes of carbon and nitrogen in the study of avian and mammalian trophic ecology. Can J Zool.

[CR19] King RA, Read DS, Traugott M, Symondson WOC (2008). Molecular analysis of predation: a review of best practice for DNA-based approaches. Mol Ecol.

[CR20] King RA, Moreno-Ripoll R, Agustí N, Shayler SP, Bell JR, Bohan DA, Symondson WOC (2011). Multiplex reactions for the molecular detection of predation on pest and nonpest invertebrates in agroecosystems. Mol Ecol Resour.

[CR21] Kremen C (2005). Managing ecosystem services: what do we need to know about their ecology?. Ecol Lett.

[CR22] Leelapaibul W, Bumrungsri S, Pattanawiboon A (2005). Diet of wrinkle-lipped free-tailed bat (Tadarida plicata Buchannan, 1800) in central Thailand: insectivorous bats potentially act as biological pest control agents. Acta Chiropterologica.

[CR23] Losey JE, Vaughan M (2006). The economic value of ecological services provided by insects. Bioscience.

[CR24] Luck GW, Daily GC, Ehrlich PR (2003). Population diversity and ecosystem services. Trends Ecol Evol.

[CR25] Luck GW, Lavorel S, McIntyre S, Lumb K (2012). Improving the application of vertebrate trait-based frameworks to the study of ecosystem services. J Anim Ecol.

[CR26] Maas B, Clough Y, Tscharntke T (2013). Bats and birds increase crop yield in tropical agroforestry landscapes. Ecol Lett.

[CR27] Markman S, Tadmor-Melamed H, Arieli A, Izhaki I (2006). Sex differences in food intake and digestive constraints in a nectarivorous bird. J Exp Biol.

[CR28] McCracken GF, Westbrook JK, Brown VA, Eldridge M, Federico P, Kunz TH (2012). Bats track and exploit changes in insect pest populations. PLoS One.

[CR29] Mendenhall CD, Sekercioglu CH, Oviedo F, Ehrlich PR, Daily GC (2011). Predictive model for sustaining biodiversity in tropical countryside. Proc Natl Acad Sci.

[CR30] Mugo HM, Kimemia JK (2011). The Coffee Berry Borer, Hypothenemus hampei Ferrari (Coleoptera: Scolytidae ) in Eastern Africa Region: The Extent of Spread, Damage and Management Systems.

[CR31] Oehm J, Juen A, Nagiller K, Neudauser S, Traugott M (2011). Molecular scatology: how to improve prey DNA detection success in avian faeces?. Mol Ecol.

[CR32] Pegard A, Miquel C, Valentini A, Coissac E, Bouvier F, François D, Taberlet P, Engel E, Pompanon F (2009). Universal DNA-based methods for assessing the diet of grazing livestock and wildlife from feces. J Agric Food Chem.

[CR33] Podulka S, Rohrbaugh RW, Bonney R (2004). Handbook of Bird Biology.

[CR34] Pompanon F, Deagle BE, Symondson WOC, Brown DS, Jarmon SN, Taberlet P (2012). Who is eating what: diet assessment using next generation sequencing. Mol Ecol.

[CR35] Schnell IB, Thomsen PF, Wilkinson N, Rasmussen M, Jensen LRD, Willerslev E, Bertelsen MF, Gilbert MTP (2012). Screening mammal biodiversity using DNA from leeches. Curr Biol.

[CR36] Sogin ML, Morrison HG, Huber JA, Welch MD, Huse SM, Neal PR, Arrieta JM, Herndl GJ (2006). Microbial diversity in the deep sea and the underexplored “rare biosphere”. Proc Natl Acad Sci U S A.

[CR37] Staver C, Guharay F, Monterroso D, Muschler RG (2001). Designing pest-suppressive multistrata perennial crop systems: shade-grown coffee in Central America. Agrofor Syst.

[CR38] Stiles FG, Skutch AF (1989). A Guide to the Birds of Costa Rica.

[CR39] Symondson WOC (2002). Molecular identification of prey in predator diets. Mol Ecol.

[CR40] Valentini A, Pompanon F, Taberlet P (2009). DNA barcoding for ecologists. Trends Ecol Evol.

[CR41] Zeale MRK, Butlin RK, Barker GL A, Lees DC, Jones G (2010). Taxon-specific PCR for DNA barcoding arthropod prey in bat faeces. Mol Ecol.

[CR42] Zuur AF, Ieno EN, Walker NJ (2009). Mixed Effects Models and Extensions in Ecology With R.

