# Supplementary material for: Molecular diagnosis of bird-mediated pest consumption in tropical farmland
Source: Springerplus. 2014 Oct 24;3:630. doi: 10.1186/2193-1801-3-630 (PMC4216319; doi:10.1186/2193-1801-3-630)

**Supplementary Tables and Figures**

**Table S1:** Generalized Linear Mixed Model (GLMM) analysis of feces derived from feeding trials (binomial error structures and logit-links). Table compares nested models through Aikaike Information Criteria (AIC) and likelihood ratio tests. The most parsimonious model is bolded.

| Response Variable | Predictor Variables | AIC | χ^2^ | *P* |
| --- | --- | --- | --- | --- |
| Detection Probability | ~#Borers+ Spp. + Time+ Weight+ #Borers:Time+ #Borers:Weight+ Time:Weight+ RE | 70.0 |  |  |
|  | ~#Borers+ Time+ Weight+ #Borers:Time+ #Borers:Weight+ Time:Weight+ RE | 68.6 | 2.59 | 0.27 |
|  | ~#Borers+ Time+ Weight+ #Borers:Weight+ Time:Weight+ RE | 67.4 | 0.83 | 0.36 |
|  | ~#Borers+ Time+ Weight+ #Borers:Weight+ RE | 67.5 | 2.11 | 0.14 |
|  | **~#Borers+ Time+ Weight+ RE** | **66.4** | **1.03** | **0.31** |
|  | ~#Borers+ Time+ RE | 79.0 | 14.9 | <0.01 |
|  | ~#Borers+ Weight+ RE | 72.9 | 8.78 | <0.01 |
|  | ~Time+ Weight+ RE | 72.1 | 7.99 | <0.01 |

# Borers= Number of borers in feeding trial; Spp= Bird species in feeding trial; Time= Elapsed time since feeding; Weight= Fecal sample weight; “:”= interaction; RE= Random effect (Feeding Trial).

**Figure S1:** Functional trait values for the six confirmed borer-consuming bird species.


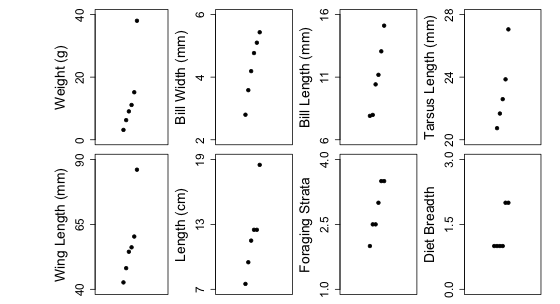

Supplement: Supplementary file 1 — Additional file 1: Supplementary Tables and Figures. (DOCX 299 KB) [file 40064_2014_1323_MOESM1_ESM.docx]
